# Automated Calibration Method for Eye-Tracked Autostereoscopic Display

**DOI:** 10.3390/s18082614

**Published:** 2018-08-09

**Authors:** Hyoseok Hwang

**Affiliations:** Department of Software, Gachon University, Seongnam, Gyeonggi-do 13120, Korea; hshwang@gachon.ac.kr; Tel.: +82-31-750-8920

**Keywords:** autostereoscopic display, automated calibration, camera calibration, eye-tracking

## Abstract

In this paper, we propose an automated calibration system for an eye-tracked autostereoscopic display (ETAD). Instead of calibrating each device sequentially and individually, our method calibrates all parameters of the devices at the same time in a fixed environment. To achieve this, we first identify and classify all parameters by establishing a physical model of the ETAD and describe a rendering method based on a viewer’s eye position. Then, we propose a calibration method that estimates all parameters at the same time using two images. To automate the proposed method, we use a calibration module of our own design. Consequently, the calibration process is performed by analyzing two images captured by onboard camera of the ETAD and the external camera of the calibration module. For validation, we conducted two types of experiments, one with simulation for quantitative evaluation, and the other with a real prototype ETAD device for qualitative assessment. Experimental results demonstrate the crosstalk of the ETAD was improved to 8.32%. The visual quality was also improved to 30.44% in the peak-signal-to-noise ratio (PSNR) and 40.14% in the structural similarity (SSIM) indexes when the proposed calibration method is applied. The whole calibration process was carried out within 1.5 s without any external manipulation.

## 1. Introduction

An autostereoscopic display is a device that allows three-dimensional (3D) perception by providing different images to each one of a person’s eyes. The biggest advantage of the autostereoscopic display is that users do not need to wear special equipment such as glasses. During the past decade, various methods have been studied to implement autostereoscopic display [1,2]. Among them, an autostereoscopic display with a conventional two-dimensional (2D) flat panel display and an additional optical layer, which is located in front or at the back of the display panel, has gained the most commercial success due to its low cost and ease of fabrication [3]. A typical method of stereoscopic display, which employs an additional optical layer, endows directionality to each pixel by blocking light paths (for parallax barriers) or by refracting light rays (for lenticular lenses). This method limits the visibility of pixels to only a specific range of view positions or angles. Located at different view positions, the left and the right eye see different sets of pixels.

Despite these advantages, several drawbacks prevent the autostereoscopic display from becoming more prevalent [4,5]. A typical method of stereoscopic display, in which the left and the right views are repeated alternately, is restricted to a specific viewing area. In the case of two views, the margin of the correct viewing area is approximately the distance between the eyes. Even if a viewer moves slightly from the fixed location, the view will transform into a depth-reversed image, referred to as a pseudoscopic image [6]. Increasing the angular resolution, i.e., the number of views, has been a promising method to overcome this problem [7]. The concept of the multiview method is based on showing the user two of several consecutive views, which have disparity over each other, instead of the left and the right views. In this case, however, the spatial resolution of each view is inevitably lowered due to the finite number of pixels. Hence, a tradeoff arises between the angular resolution and the spatial resolution. In addition, complete separation between all views is challenging, so there is a crosstalk where several views overlap with each other.

To solve these problems, an eye-tracked autostereoscopic display (ETAD) has been proposed [8,9,10,11]. Instead of increasing the number of views, the ETAD uses the left and right views only. To prevent the user’s eyes from moving out of the correct viewing area, the ETAD employs an additional device, e.g., a camera or a depth sensor, for detecting and tracking the eye position of a viewer. Using the eye tracking device, the ETAD ensures that the user’s eyes are always in the correct viewing zones by moving the optical element mechanically [12], by moving slits on an additional LC display [13,14], or by merging viewing zones into two views around the viewer’s eyes [6].

To date, there has been various studies to improve the visual quality of the ETAD, which mainly focus on minimizing crosstalk [4], reducing visual fatigue [15], or estimating the accurate position of a viewer [16]. These studies implicitly assumed that all parameters of the ETAD are known accurately or are the same as the designed parameters. In practice, however, parameters of the ETAD are likely to differ from the designed values for many reasons, especially due to inaccuracy in the production and the assembly processes. Moreover, the ETAD system, which employs additional devices and processes, requires parameters that are more accurate because the accumulation of errors from each device significantly degrades the visual quality. Therefore, parameters of the ETAD must be calibrated prior to the operation.

Several studies have investigated the calibration of the ETAD. Sandin et al. [17] described a calibration method of Varrier^TM^, which consists of multiple autostereoscopic displays with a stereo camera for user’s head tracking. To calibrate each optical element of the displays, they used external stereo cameras. However, they did not describe the method for calibrating the pose between the head tracking camera and the coordinates of each device. Bailer et al. [18] proposed a calibration method for a moving barrier type ETAD system. In the calibration process, they mapped each pixel on a captured camera image to the pixels of a display panel. This method is simple to implement; however, the calibration process should be conducted in a dark room. Moreover, the mapping pixels between the image and the panel are only valid when the viewer is at a certain distance. In the case of Nintendo^TM^ 3DS, which is commercially available, the calibration is carried out by the user [19]. A user continuously changes parameters while looking at the screen until the optimum visual quality is achieved.

Thus far, there has been no method to calibrate all parameters of the devices consisting the ETAD. In calibrating the ETAD, therefore, the most suitable method to date is to calibrate each device individually and sequentially. Below, we present a calibration example for an ETAD consisting of an onboard camera for eye tracking, a 2D display panel, and an optical element such as lenticular lenses or parallax barriers. First, we calibrated an onboard camera by analyzing several images, which capture patterns of various postures in front of the camera [20]. Then, we calibrated the optical element by the visual pattern analysis method [21]. This method uses two images of the display, thus requiring an external camera setup. Finally, the extrinsic pose between the onboard camera and the display panel should be calibrated. There are few studies on calibrating the extrinsic pose of these two devices. The second-best method is to apply a similar approach as in [22], in which the extrinsic pose of the camera and the pattern on a robot are calibrated. Even though we employed this method, problems still exist. To calibrate the pose of the camera and the pattern looking in the same direction, a mirror is needed. Furthermore, several movements of the device are required to capture images at various angles. Applying this approach to the ETAD can lead to a serious problem: parameters of the device may be changed by repeated movements of the device. In addition, individual calibration methods have a serious problem. The calibration errors of each device are accumulated and finally affect to the visual quality. The sequential approach described above is also not suitable for automated calibration. For automated calibration, an auxiliary manipulator is needed to move calibration patterns or the ETAD itself. In terms of mass production, this method increases both cost and processing time of the device calibration.

For automated calibration of the ETAD, several problems should be solved. It has not been clearly established which parameters of the ETAD need to be calibrated. A novel methodology would be to calibrate all of these parameters not individually but simultaneously for cost reduction. To alleviate parameter change during the calibration process, the method should minimize manipulation or even calibrate in a fixed environment. In this paper, we introduce an automated calibration method for the ETAD. The main contributions of this study are as follows:

- We establish the parameters that require calibration. To achieve this, we firstly address the process of the ETAD from eye tracking to 3D image rendering. To the best of our knowledge, the whole process of the ETAD has never been explored before.
- We propose a novel methodology, which calibrates all devices of the ETAD. Our calibration approach is mainly focused on both the automated and the simultaneous calibration. This method calibrates all parameters at the same time, not sequentially. The proposed method also does not require the movement of the ETAD or the calibration device, but instead is performed in a fixed environment. To achieve this goal, we designed and implemented a calibration module that consists of a 3D pattern and an external camera. The module enabled us to calibrate an ETAD by simply setting it in front of the calibration module.

The rest of the paper is organized as follows. In Section 2, we establish the parameters of the ETAD by addressing its physical model and rendering process. Section 3 presents a calibration method, which estimates all parameters automatically and simultaneously. Then, we experimentally validate the proposed method in Section 4. Finally, Section 5 concludes the paper.

## 2. Parameters Establishment

In this section, we establish the parameters of the ETAD that need to be calibrated in two stages. First, we describe the parameters of the devices that comprise a conventional ETAD. Here, the conventional ETAD refers to an autostereoscopic device having a camera for eye tracking and an additional optical layer, e.g., lenticular lenses or parallax barriers, on a 2D flat panel. Note that parameters are common even though the types of optical layers are different. Then, we describe the rendering process of the ETAD in order to identify the parameters.

### 2.1. Physical Model of the ETAD

**Figure 1:**
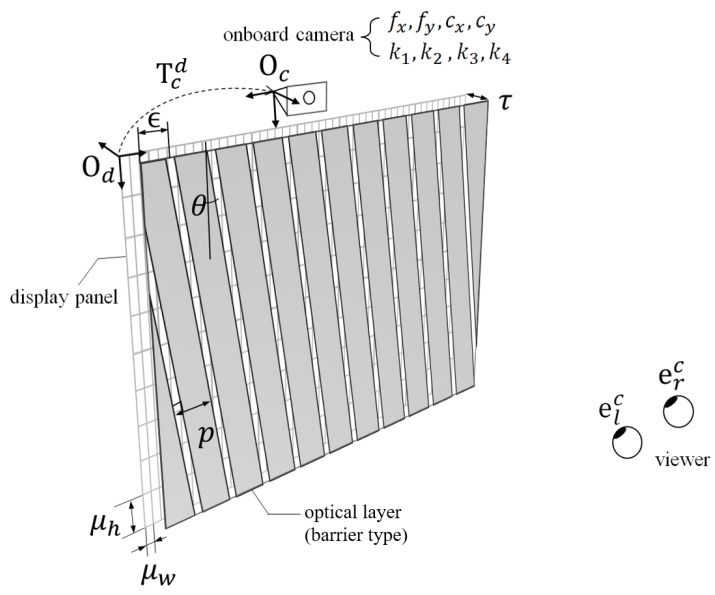
Parameters of an Eye-Tracked Autostereoscopic Display (ETAD).

The parameters of the conventional ETAD devices are represented in Figure 1. The conventional ETAD consists of three modules: a two-dimensional flat panel, an optical layer, and an eye-tracking sensor. The two-dimensional flat panel is a collection of pixels and each pixel has a width $\mu_{w}$ and height $\mu_{h}$. Most of optical layers are lenticular lenses or parallax barriers [23]. In addition, these two optical layers have common parameters. A pitch *p* represents the distance between the lens in the case of lenticular lenses and between the slits in the case of parallax barriers. The slanted angle θ represents the degree of tilting of the optical layer. The gap τ is the distance of the slit of the barrier or the focal plane of the lens from the flat panel. The offset parameter η represents the horizontal translation of the optical layer from the origin of the display coordinate system. An onboard camera is attached at the top of the display panel for tracking a viewer’s eyes. To reconstruct the three-dimensional position from a point on an image, intrinsic parameters and distortion parameters of the camera are used. The intrinsic parameters include focal lengths $f_{x},f_{y}$ and principal points $c_{x},c_{y}$. The parameters $k_{1},k_{2},k_{3},$ and $k_{4}$ denote distortion coefficients. There are two coordinate systems: the onboard camera coordinate system and the display panel coordinate system.

### 2.2. Rendering Process

Basically, the rendering process for the ETAD is considered as an iterative process comprising two steps: the eye position estimation step and the image-multiplexing step. In the eye position estimation step, the system estimates the 3D position of the eyes from the image captured by the onboard camera. Note that the eye detection method is beyond the scope of this study. In the image-multiplexing step, the system generates a multiplexing image according to the eye positions. Let us describe the iterative process systematically.

**Eye Position Estimation Step:** Given the eye positions on a captured image from the onboard camera, they are derived in the camera coordinate system as

(1) $$\left[\begin{array}{c} x_{\mu }\\ y_{\mu } \end{array}\right]=\left[\begin{array}{c} \frac{s_{\mu }-c_{x}}{f_{x}}\\ \frac{t_{\mu }-c_{y}}{f_{y}} \end{array}\right],$$

where $s_{\mu }$ and $t_{\mu }$ are eye positions on the captured image and $x_{\mu },y_{\mu }$ are the corresponding positions on the onboard camera coordinate system. Note that the subscript μ is either left or right $\left(\mu \in \left\{l,r\right\}\right)$ and the equation containing μ is the same in the left and right cases. The parameters $f_{x},f_{y}$ are focal lengths in the *x* and *y* directions, respectively, and $c_{x},c_{y}$ are principal points of the onboard camera. Commercial camera lenses conventionally have distortion, known as radial and tangential distortion [24]. We define $k_{1},k_{2}$ as the parameters for radial distortion, and $k_{3},k_{4}$ as the parameters for tangential distortion of the camera lens. Using these distortion parameters, the undistorted position of each eye $x_{\mu }^{\prime },y_{\mu }^{\prime }$ is derived as

(2) $$\left[\begin{array}{c} x_{\mu }^{\prime }\\ y_{\mu }^{\prime } \end{array}\right]=\left(1+k_{1}r^{2}+k_{2}r^{4}\right)\left[\begin{array}{c} x_{\mu }\\ y_{\mu } \end{array}\right]+\left[\begin{array}{c} k_{3}\left(2x_{\mu }y_{\mu }\right)+k_{4}\left(r^{2}+2{x_{\mu }}^{2}\right)\\ k_{3}\left(r^{2}+2{y_{\mu }}^{2}\right)+k_{4}\left(2x_{\mu }y_{\mu }\right) \end{array}\right],$$

where $r_{\mu }=\sqrt{x_{\mu }^{2}+y_{\mu }^{2}}$. Then, we can obtain the ray vectors, which have direction information of both eyes from the origin of the camera coordinate system. The ray vector $v_{\mu }$ which passes through $x_{\mu }^{\prime },y_{\mu }^{\prime }$ from the origin of the camera coordinate system $O_{c}$ can be represented in homogeneous coordinates as

(3) $$v_{\mu }=\left[\begin{array}{c} x_{\mu }^{\prime }\\ y_{\mu }^{\prime }\\ 1 \end{array}\right].$$

Given these back-projected vectors, we finally obtain the three dimensional eye positions $e_{l}^{c}$, $e_{r}^{c}$ as

(4) $$e_{l}^{c}=\frac{\psi }{∥v_{l}-v_{r}/\alpha ∥}v_{l},$$

(5) $$e_{r}^{c}=\frac{\psi }{∥\alpha v_{l}-v_{r}∥}v_{r},$$

where α is defined as

(6) $$\alpha =\frac{v_{r}^{T}\phi }{v_{l}^{T}\phi },$$

Here, we assume that interpupillary distance (IPD) ψ is known and the direction of the face normal vector ϕ faces the onboard camera. The proof for Equations (4) and (5) are given in Appendix A at the end of the paper.

**Image Multiplexing Step:** In our rendering approach, we can simplify the method of generating a multiplex image as a problem of assigning labels (left or right) to each pixel. Note, a pixel generally consists of several sub-pixels. However, here we define the pixels in the smallest unit, i.e., sub-pixels, as “pixels”. The multiplexed image is generated from the number of viewpoint images, e.g., a two-view display requires two images: the left and the right image. As described above, all pixels have directionality by the additional optical layer. Pixels are assigned the “left” label to show the same intensity value of the left source image if their direction is toward the left eye of the viewer; the same condition is applicable to the “right” case. On the contrary, we can assign a label to each pixel by determining whether the pixel’s position is close to the visible pixels of the left eye or those of the right eye. The slits of the barrier or principle points of a lenticular lens can be expressed as a set of lines; therefore, the pixels passing through the slit and directed to the left or right eye are also represented by lines. Here, we would like to define these sets of lines as the visible pixels of the left eyes and those of the right eyes. Then, we assign a label value that corresponds to the nearest visible line for the all pixels, not visible directly.

The 3D eye position $e_{\mu }^{c}$ obtained from the captured image is based on the camera coordinate system; we can convert it into the display coordinate system as

(7) $$\left[\begin{array}{c} e_{\mu }^{d}\\ 1 \end{array}\right]=T_{c}^{d}\left[\begin{array}{c} e_{\mu }^{c}\\ 1 \end{array}\right]$$

where $T_{c}^{d}$ is a homogeneous transform matrix from an onboard camera coordinate system to a display coordinate system. The transform matrix can be represented as

(8) $$T_{c}^{d}=\left[\begin{array}{cc} R_{c}^{d} & t_{c}^{d}\\ 0 & 1 \end{array}\right],$$

which consisting of the transform matrix $t_{c}^{d}={\left[\begin{array}{ccc} t_{x} & t_{y} & t_{z} \end{array}\right]}^{T}$ and the rotation matrix $R_{c}^{d}$. The rotation matrix is defined as

(9) $$R_{c}^{d}=\left[\begin{array}{ccc} \cos r_{z}\cos r_{y} & \cos r_{z}\sin r_{y}\sin r_{x}-\sin r_{z}\cos r_{x} & \cos r_{y}\sin r_{y}\cos r_{x}+\sin r_{z}\sin r_{x}\\ \sin r_{z}\cos r_{y} & \sin r_{z}\sin r_{y}\sin r_{x}+\cos r_{z}\cos r_{x} & \sin r_{x}\sin r_{y}\cos r_{z}-\cos r_{z}\cos r_{x}\\ -\sin r_{y} & \cos r_{y}\sin r_{x} & \cos r_{y}\cos r_{x} \end{array}\right],$$

where $r_{x},r_{y},r_{z}$ are Euler angles in radians.

We define the *n*th line of the slits of an optical element as $S_{n}$; the vector equation is represented as

(10) $$S_{n}=\left[\begin{array}{c} \epsilon +\frac{p}{\cos\theta }n\\ 0\\ \tau \end{array}\right]+\delta \left[\begin{array}{c} \tan\theta \\ 1\\ 0 \end{array}\right],$$

where ϵ is the offset, θ is the slanted angle, p/cosθ is the horizontal pitch, and τ is the gap of the optical layer. The left term of this equation represents the origins of slits, and the right term the directional vector with an arbitrary real number δ. We obtain the positions of the visible pixel $s_{n,\mu }^{\prime }$ which is represented as the projected vectors on the panel using the eye positions and the slit vectors as

(11) $$S_{n,\mu }^{\prime }=\frac{1}{z_{\mu }-\tau }\left(z_{\mu }\left[\begin{array}{c} \epsilon +\frac{p}{\cos\theta }n\\ 0\\ \tau \end{array}\right]-\tau e_{\mu }^{d}\right)+\delta \left[\begin{array}{c} \tan\theta \\ 1\\ 0 \end{array}\right],$$

where $z_{\mu }\left(\mu \in l,r\right)$ is the distance to the left or the right eye. Then, we also express a pixel position of u,v on the display panel as

(12) $$P_{u,v}=\left[\begin{array}{c} u\lambda_{w}\\ v\lambda_{h}\\ 0 \end{array}\right],$$

where $\lambda_{w},\lambda_{h}$ are the width and height of a pixel, respectively.

We obtain the distances between a pixel P and all visible pixels $s_{n,\mu }^{\prime }$ by the function *D*, which is given by

(13) $$D\left(P_{u,v},S_{n,\mu }^{\prime }\right)=\frac{\left(P_{u,v}-\frac{1}{z_{\mu }-\tau }\left(z_{\mu }\left[\begin{array}{c} \epsilon +\frac{p}{\cos\theta }n\\ 0\\ \tau \end{array}\right]-\tau e_{\mu }^{d}\right)\right)\times \left[\begin{array}{c} \tan\theta \\ 1\\ 0 \end{array}\right]}{∥\left[\begin{array}{c} \tan\theta \\ 1\\ 0 \end{array}\right]∥},$$

where × represents the cross product and $∥\cdot ∥$ denotes the two-norm.

Finally, we determine the label of the pixel by comparing it with the minimum distances to the left and the right visible pixels as

(14) $$L\left(u,v\right)=\left\{\begin{array}{ll} “\mathrm{left}”, & \min\left(D\left(P_{u,v},S_{n,l}^{\prime }\right)\right)<\min\left(D\left(P_{u,v},S_{n,r}^{\prime }\right)\right)\\ “\mathrm{right}”, & \mathrm{otherwise} \end{array}\right..$$

**Figure 2:**
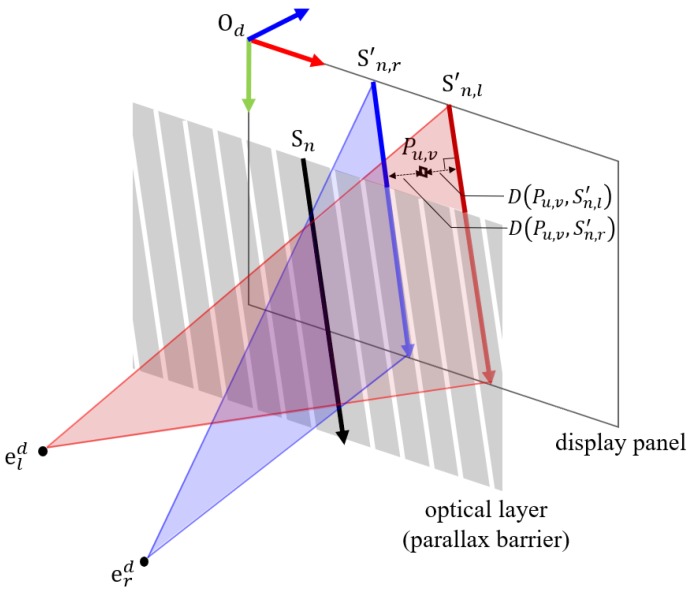
Overview of calibration process using the calibration module. The calibration module consists of a three-dimensional calibration pattern and an external camera.

The pixels with labels “left” have the pixel intensity of those positions on the left source image; the same applies for the “right” label case. An overview of the calibration process is presented in Figure 2.

### 2.3. Parameter Classification

As described above, various parameters are required to generate a correct multiplex image for a certain viewing position. This means that accumulation of small parameter errors can cause visual quality degradation; therefore, accurate calibration is needed for the ETAD. The parameters are listed in Table 1. We classify these parameters into two categories: constant and variable parameters. Constant parameters have absolute values, determined when manufacturing the devices and there is little variation between the devices. These constant parameters also hardly change with movement. Therefore, it is sufficient to use designed parameters directly for the constant parameters. The width and height of the pixel $\mu_{w}$, $\mu_{h}$, and the pitch of an optical layer *p* belong to this category.

On the other hand, some parameters, classified as variable parameters, are not only different from the design value, but even have different values for each ETAD. These errors mainly occur during the production process of each device. These errors can even increase if the device is not fixed during the calibration process. For onboard cameras, focal lengths and principal points, which are dependent on the pose of the image sensor and the lens, vary from device to device. The radial distortions of cameras also have different values between each other. Some parameters of the additional optical layers, such as the slanted angle, the gap, and the offset, are determined in the assembly process with the display panel, because those parameters are relative to the display coordinates. The relative pose between the onboard camera coordinate system and the display coordinate system also depends on how the onboard camera is attached to the display. In our study, therefore, we focus on the calibration of those “variable parameters”. Based on the classification of parameters, we describe the calibration method in the next section.

## 3. Calibration

### 3.1. Calibration Process

The proposed method uses two images to calibrate all the parameters of the ETAD. One image is for estimating the parameters of the onboard camera and the other image is for parameters of the optical layer. To estimate camera parameters, i.e., intrinsic parameters and distortion coefficients, the onboard camera takes a picture of the calibration pattern. The same applies to calibrating the optical layer. In this case, the optical layer is a kind of pattern to be captured. Because the viewing angle of the onboard camera does not include the optical layer, an additional camera is required to capture the optical layer. In summary, we use two different cameras: the onboard camera and the external camera; hence, we can capture two images at the same time. To estimate the pose between the onboard camera and the display panel, we introduce an indirect pose estimation method. As mentioned above, it is not possible to directly capture the display panel or the optical layer with only the onboard camera. Some methods use equipment, e.g., mirrors, to capture objects to estimate the pose. These methods require several images, so a robot or a manipulator that can move the mirror is needed. Alternatively, the object in front of the mirror should be moved, which would cause parameter changes. Our method estimates the final pose by combining three poses. Two poses are estimated from two captured images and have different values for each calibration. The other pose is estimated once and is fixed.

**Figure 3:**
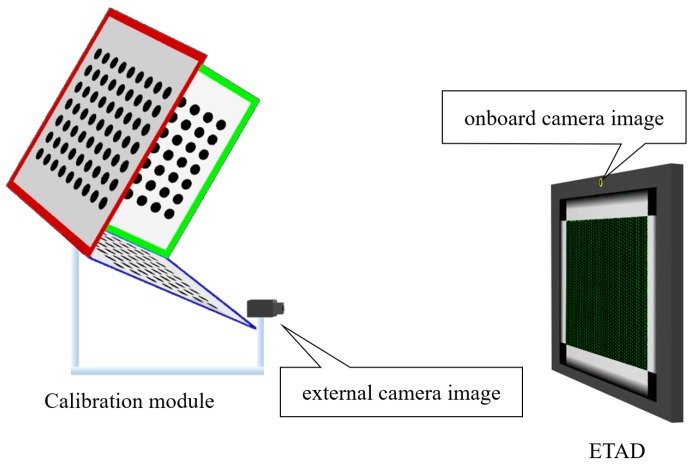
Overview of the calibration process using the calibration module. The calibration module consists of a 3D calibration pattern and an external camera.

With the proposed calibration method, we can calibrate the ETAD automatically and simultaneously; however, additional elements are needed: the calibration pattern for the onboard camera and the external camera to capture the display panel. We designed a calibration module that consists of these two devices: the calibration pattern and the additional camera, which has a common structure with the ETAD (see Figure 3). As with the ETAD, the calibration pattern is not included in the viewing angle of the external camera of the calibration module. Therefore, we placed the ETAD in front of the calibration module for the calibration process. The onboard camera of the ETAD captures an image of the calibration pattern of the calibration module. Oppositely, the external camera of the calibration pattern captures an image of the optical layer with the display pattern. Here, we can estimate the parameters of the ETAD devices, i.e., the onboard camera and the optical layer, because we know in advance the parameters of the devices constituting the calibration module.

**Figure 4:**
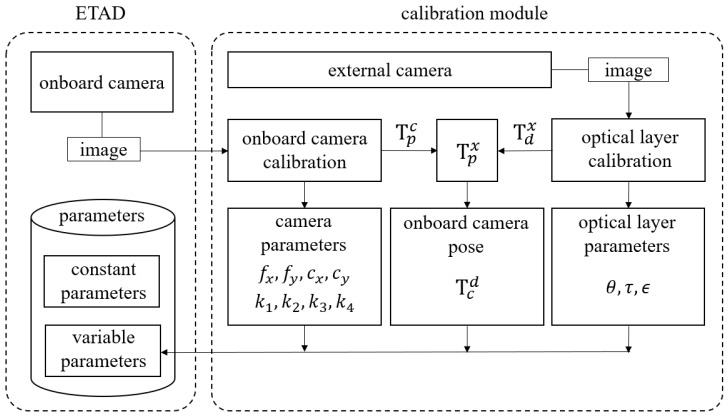
Block diagram of the calibration process.

The block diagram of the calibration process with two images is represented in Figure 4. When the calibration begins, the onboard camera and the external camera simultaneously capture the patterns on the opposite side. Using the onboard camera image, the system calibrates the onboard camera parameters, i.e., $f_{x},f_{y},c_{x},c_{y},k_{1},k_{2},k_{3},k_{4}$. At this time, it also estimates the transformation matrix $T_{c}^{p}$ from the calibration pattern coordinate system to the onboard camera coordinate system. Simultaneously, the system calibrates the optical element parameters, i.e., θ,τ,ϵ, using the external camera image. The onboard camera pose, i.e., $r_{x},r_{y},r_{z},t_{x},t_{y},t_{z}$, which also can be represented as the transformation matrix $T_{c}^{d}$ is finally calibrated using two estimated transformation matrix $T_{c}^{p},T_{d}^{x}$ and $T_{x}^{p}$ calibrated in advance. The details of the calibration method are presented in the following section.

### 3.2. Calibration Method

**Onboard camera calibration:** The onboard camera parameters are calculated by analyzing the pattern image of the 3D calibration pattern of the calibration module. We employ Zhang’s camera calibration method [20] to estimate both intrinsic parameters and distortion parameters of the onboard camera. The calibration pattern of the calibration module is built such that the three 2D patterns are orthogonal to each other. Hence, once an image is captured, the system detects a 3D pattern on the image and split it into three 2D patterns. However, three images are not enough to estimate accurate parameters using [20]; therefore, we add an orthogonal constraint among the estimated poses of the three planes. Using the known 3D points and the corresponding points in the captured image, we estimate the onboard camera parameters and the poses of the plane patterns by minimizing the following functional:

(15) $$\sum_{i=1}^{3}\sum_{j=1}^{m}{∥m_{ij}-\overset{˘}{m}\left(f_{x},f_{y},c_{x},c_{y},k_{1},k_{2},k_{3},k_{4},R_{i},t_{i},M_{j}\right)∥}^{2}-\lambda ∥{\hat{R}}^{T}\hat{R}-I∥$$

where the left term denotes reprojection error and the right term denotes the orthogonal constraint term. In the left term, *j* represents the index of the points and *i* represents the index of the 2D patterns. The function $\overset{˘}{m}$ projects the 3D point $M_{j}$ of the *i*th pattern, and $m_{ij}$ corresponds to the points on the image. In the right term, λ is a weight factor; we set this term to 0.01. The matrix $\hat{R}$ is defined by a composition of vectors, which are the basis vectors of $R_{1},R_{2},R_{3}$ and can be represented as

(16) $$\hat{R}=R_{1}\left[\begin{array}{ccc} 1 & 0 & 0\\ 0 & 0 & 0\\ 0 & 0 & 0 \end{array}\right]+R_{2}\left[\begin{array}{ccc} 0 & 0 & 0\\ 0 & 1 & 0\\ 0 & 0 & 0 \end{array}\right]+R_{3}\left[\begin{array}{ccc} 0 & 0 & 0\\ 0 & 0 & -1\\ 0 & 0 & 0 \end{array}\right].$$

**Figure 5:**
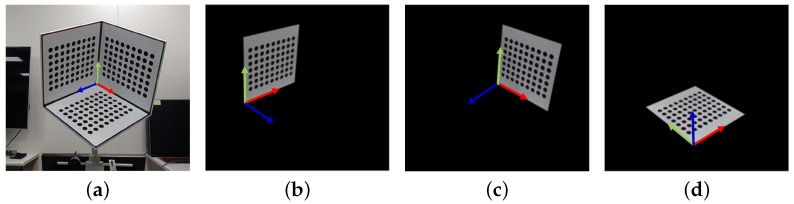
Onboard camera parameter calibration using a 3D calibration pattern of the calibration module: (**a**) onboard camera image; (**b**) the first 2D calibration pattern; (**c**) the second 2D calibration pattern; and (**d**) the third 2D calibration pattern. The red, green, and blue arrows represent the x-, y-, and z-axis, respectively.

Because the three planes of the calibration pattern are orthogonal, the matrix $\hat{R}$ also has to satisfy orthogonality. This means that the product of $\hat{R}$ and the transpose of $\hat{R}$ is the identity matrix I. The optimization is solved using Levenberg–Marquardt nonlinear optimization [25]. The transformation matrix $T_{x}^{c}$ can be estimated as

(17) $$T_{x}^{c}=\left[\begin{array}{cc} R_{2} & t_{2}\\ 0 & 1 \end{array}\right],$$

because the coordinate system of the second pattern is the same as the 3D calibration pattern coordinate system (see Figure 5).

**Optical layer calibration:** The system calibrates the parameters of the optical layer such as the pitch, the slanted angle, the gap, and the offset. Our method is mainly based on the visual pattern analysis method [21], which uses two captured images by an external camera. First, a pattern is displayed on the panel. This pattern is composed of two vertically striped patterns to avoid ambiguity between the slanted angle and the pitch. The two vertical stripe patterns have different distances and different colors, e.g., blue and green, to separate them later. However, the ambiguity between the gap and the pitch remains. To solve this problem, Hwang et al. [21] captured the pattern images twice at different distances. They first capture the pattern image at a close distance, then move the camera and capture the image from a long distance. It is not suitable to apply this method directly to our system because of the movement of the camera. We propose a revised method to calibrate the parameters of the optical layer in a fixed environment. The key idea of our method is that we only take into account three parameters of the optical layer, excluding the pitch because it was classified as a constant parameter. With the revised method, ambiguities are solved and only one capture image of the pattern is required to calibrate parameters.

We first displayed the pattern image on the display panel and capture the image using the external camera. We employed a state-of-the-art method to estimate camera pose called homography decomposition [26]. We detected positions of the four corners of the display in the captured image and found homography between the external camera coordinates and the image coordinates. The homography can be decomposed into the intrinsic parameters of the external camera and the camera pose matrix, which is denoted as $T_{x}^{d}$ in this paper. We assumed the intrinsic parameters of the extrinsic camera are calibrated in advanced to this process, thus the camera pose matrix $T_{x}^{d}$ can be computed by multiplying the inverse of the intrinsic matrix to the homography. Even if we found the four corners of the pattern area, it is necessary to restore the pattern to its original shape because the shape of the pattern in the captured image may appear distorted due to the camera rotation. To calibrate the system with the captured image, a rectification process is needed. We used a simpler scheme that warps the four corners to the vertices of a rectangle of the display pattern [24].

**Figure 6:**
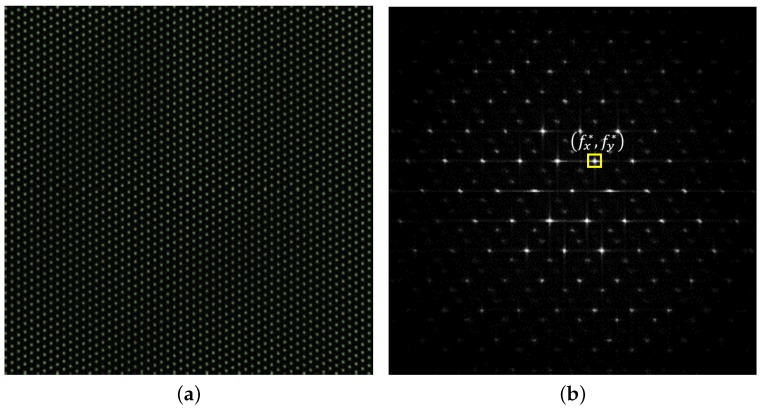
The captured lattice pattern is represented in: (**a**) spatial domain; and (**b**) frequency domain. $\left(f_{x}^{*},f_{y}^{*}\right)$ on the frequency domain is the peak point.

In the captured image, the original stripe pattern is shown as a lattice pattern (see Figure 6a). This lattice pattern is generated by the intersection of the two stripes: the stripes of the display pattern and the stripes of the optical layers. The lattice pattern *L* can be represented as

(18) $$L\left(x,y\right)=\sum_{m=-\infty }^{\infty }\sum_{n=-\infty }^{\infty }\left\{\delta \left(x-m\beta \right)\cdot \delta \left(y-\frac{1}{\tan\theta }\left(m\beta -n\frac{dp}{(d-\tau )\cos\theta }\right)\right)\right\},$$

where δ denotes Dirac’s delta function. In Equation (18), x,y represent the positions on the lattice pattern, and β denotes the horizontal space of the display pattern. The distance of the external camera from the display pattern is represented as *d*. Note, the parameter beta is fixed and the distance *d* was obtained when estimating the external camera pose. Therefore, we can estimate parameters of the optical layer p,τ by analyzing the positions of x,y and then determining optimal values of m,n. Generally, a 2D lattice in a spatial domain is mapped to another 2D lattice in the frequency domain [27]. In the frequency domain, Equation (18) is transformed into

(19) $$\hat{L}\left(f_{x},f_{y}\right)=C\sum_{m=-\infty }^{\infty }\sum_{n=-\infty }^{\infty }\left\{\delta \left(f_{x}-m\frac{1}{\beta }+n\frac{(d-\tau )\cos\theta }{dp}\right)\cdot \delta \left(f_{y}-n\tan\theta \frac{(d-\tau )\cos\theta }{dp}\right)\right\},$$

where *C* denotes a constant scale factor. Estimating a peak point $\left(f_{x},f_{y}\right)$ of the lattice in the frequency domain instead of (x,y) in the spatial domain has several advantages. The signal-to-noise ratio (SNR) around the peak is quite high in the frequency domain; therefore, the position of the peak point is estimated more accurately. Moreover, noise is well suppressed in the frequency domain [21].

For estimating the parameters of the optical layers, we first transformed the warped pattern image I(x,y), which is in the form of a 2D lattice in a spatial domain to a frequency domain image $\hat{I}\left(f_{x},f_{y}\right)$ using the discrete Fourier transform. Then, we estimated a peak point of the lattices in the frequency domain. To estimate the accurate peak position, the paraboloid fitting was exploited. The parameters concerned with the display pattern are already known, thus the parameters of the optical layer can be estimated by decoding the lattice pattern. After detecting the peak point $f_{x}^{*},f_{y}^{*}$ in the frequency domain, the number of stripes *m* and the number of slits *n* are estimated as

(20) $$\left(m^{*},n^{*}\right)=\arg\min_{m,n}\left|p_{0}-\frac{d-\tau_{0}}{d}\left(\frac{n}{m/\beta -f_{x}^{*}}\right)\cos\left(\arctan\frac{f_{y}^{*}}{m/\beta -f_{x}^{*}}\right)\right|,$$

where $p_{0}$ and $\tau_{0}$ mean the designed pitch and gap, respectively. The selected *m* and *n* bring the estimated pitch closer to the designed one. We determined $m^{*}$, $n^{*}$ by exhaustive search within the range 1–20. The slanted angle θ and the gap τ are then derived using the peak points $f_{x},f_{y}$ and the selected numbers of $m^{*},n^{*}$ as

(21) $$\theta =\arctan\frac{f_{y}^{*}}{m^{*}/\beta -f_{x}^{*}},$$

(22) $$\tau =z\left(1-\frac{p}{\cos\theta }\frac{m^{*}/\beta -f_{x}^{*}}{n^{*}}\right).$$

Finally, we generated a lattice pattern using these parameters with zero offset. The offset ϵ of the optical element was estimated by measuring the vertical shift between the generated lattice pattern and the captured pattern.

**Figure 7:**
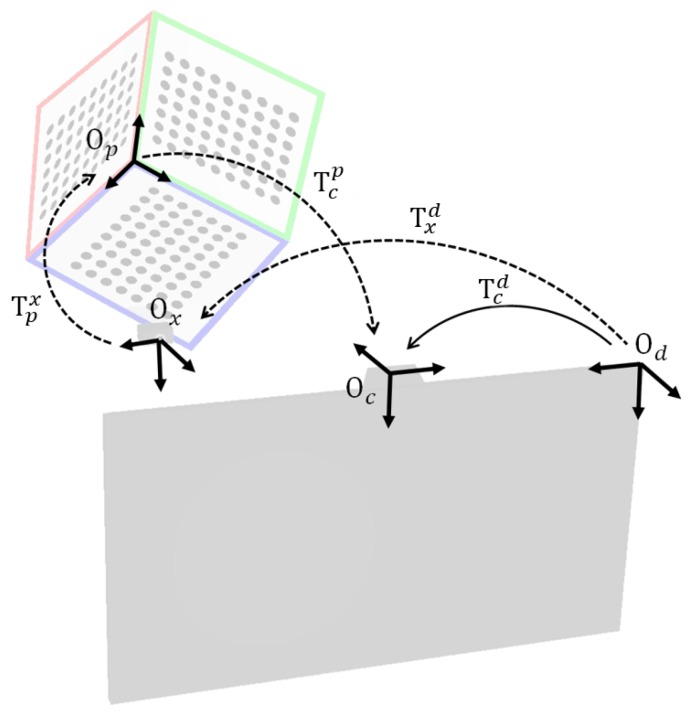
Coordinate systems with respect to each device of the ETAD. The arrow with solid line is the direct pose estimation. The arrows with dashed lines are paths of indirect pose estimation.

**Onboard camera pose calibration:** The onboard camera pose plays an important role because it converts the three-dimensional position of the viewer’s eyes from the camera coordinate system to the display coordinate system. For calibrating the onboard camera pose, we propose an indirect pose estimation method instead of direct pose estimation. As mentioned earlier, the direct pose estimation has several problems and is not suitable for our method, which aims toward an automated and simultaneous calibration process. With the indirect pose estimation method, we estimated the pose of the onboard camera $T_{c}^{d}$ by multiplying several poses as

(23) $$T_{c}^{d}=T_{x}^{d}T_{p}^{x}T_{c}^{p},$$

where $T_{x}^{d}$ and $T_{c}^{p}$ are the inverse matrix of $T_{d}^{x}$ and $T_{p}^{c}$. $T_{p}^{x}$ denotes the pose between the external camera and the calibration pattern on the calibration module (see Figure 7). Therefore, once the $T_{p}^{x}$ is estimated in advanced to the calibration process, it can be used as a fixed value each time the ETAD is calibrated. The other poses, such as $T_{x}^{d}$ and $x_{p}T_{c}^{p}$, have different values for each calibration process of the ETAD. Note, however, that the estimation of these poses is not needed because they are already estimated in the process of calibrating other devices. $T_{c}^{p}$, which denotes the pose between the onboard camera and the calibration pattern, is estimated in the process of the onboard camera calibration. Likewise, the pose between the display coordinate system and the calibration pattern coordinate system $T_{x}^{d}$ is estimated in the optical layer calibration process. Therefore, there are no additional processes for estimating the onboard camera pose without multiplying the already known pose matrix.

The proposed indirect method has two advantages. First, the calibration process performed in this method is carried out under a fixed environment. Second, no additional devices or processes are required for estimating the onboard camera calibration, which significantly reduces the calibration time of the ETAD.

## 4. Experiments and Discussion

We conducted two types of experiments to evaluate the performance of the proposed calibration scheme. First, we evaluated our method on a synthetic dataset by using a simulation tool. The simulation provides a controlled environment, i.e., we set up the system and obtained images with various parameters. In this case, the ground-truth parameters are available for the synthetic dataset; therefore, we could quantitatively measure the estimation error. We also conducted the experiment in the same manner by adding noise to the original dataset and evaluated the robustness of the proposed method.

**Figure 8:**
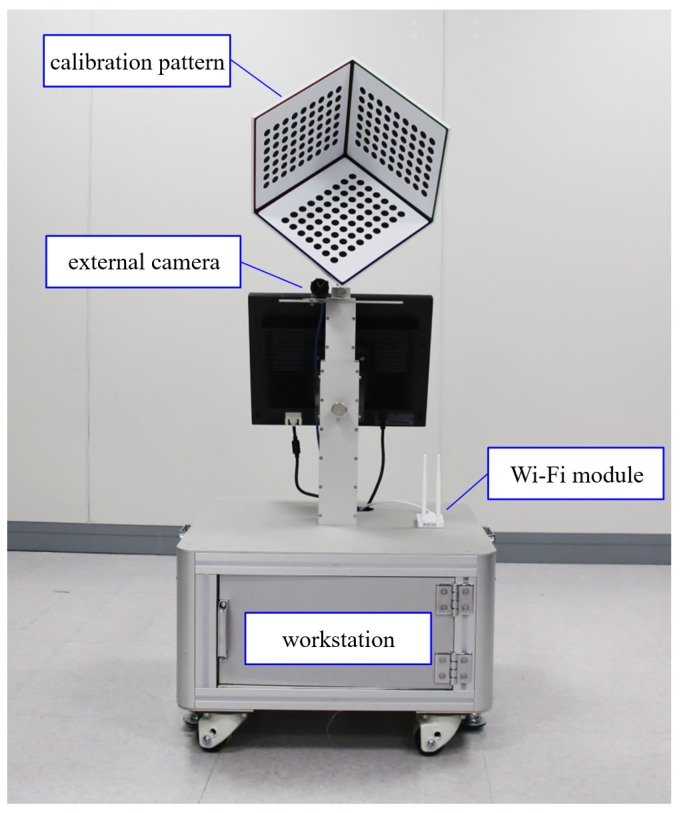
Calibration module.

Next, we applied the calibration scheme to real-life displays. We used the calibration module that we designed and built as described in the previous section (see Figure 8).

The calibration module contains not only a calibration pattern and an external camera but also a workstation and a Wi-Fi module. Through Wi-Fi, the ETAD transmits the image of the onboard camera to the workstation and receives the calibration results. The proposed calibration algorithms were implemented on a workstation equipped with an Intel Core(TM) i7-4960 processor and 64 GB of memory. In the case of the real-life ETAD, the actual parameters were relatively different from the designed ones. However, we could not measure the errors directly because the ground-truth parameters were unknown. We evaluated the performance in the visual aspect of the observed images. We prepared a measuring device that mimics a person: a fake face was attached on the stereo cameras. The ETAD continuously rendered 3D images according to the position of the viewer’s eyes. The stereo camera captured the left image and the right image. With the captured images, we assessed the visual quality with two types of evaluation: the first was the measurement of the crosstalk using red-blue images and the second was the image quality measurement using PSNR and SSIM index [28].

### 4.1. Simulation Environment

For the synthetic dataset, we built a simulation environment based on a ray-tracing tool (POV-RAY [29]). The first system was built using the designed parameters listed in Table 2. We generated 100 datasets by adding or subtracting random values from the designed ones, except for the pixel width, pixel height, and pitch parameters, which are classified as constant. We also used constant values for principal points and distortion coefficients for the onboard camera because they cannot be handled with the simulation tool. In the calibration evaluation, however, few attempts were made to measure errors of parameters directly instead of measuring the standard deviation or reprojection error. Thus, we used fixed values for the principal points of the onboard camera ($c_{x}=960,c_{y}=480$) and set all of the distortion coefficients ($k_{1}$–$k_{4}$) to zero. We defined the resolution of the display panel as 1920 × 1.080 and both pixel sizes $\mu_{x},\mu_{y}$ as 0.0846 mm. For each case of the datasets, we captured two images, one from the position of the onboard camera and the other from positions of the external camera of the calibration module. Given the two captured images, we first simultaneously estimated the onboard camera parameters and pose of the onboard camera, as well as the optical layer parameters and the pose of the display panel. Then, we finally estimated the onboard camera pose parameters from the origin of the display panel. The total processing time was 0.8 s. The estimation results are presented in Table 2. The accuracy of the focal length is comparable to that of the existing camera calibration method [30], by comparing the standard deviation.

**Figure 9:**
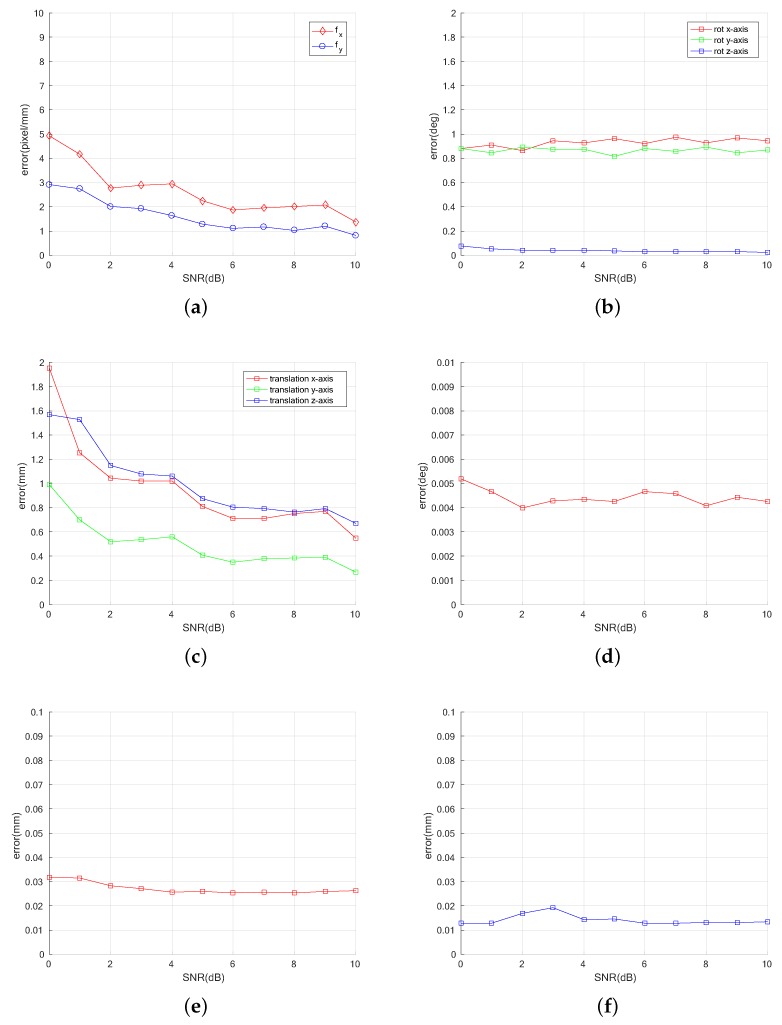
Mean absolute error for parameter estimation in noisy settings: (**a**) focal length $f_{x},f_{y}$ of the onboard camera; (**b**) rotational error $r_{x},r_{y},r_{z}$; (**c**) translational error $t_{x},t_{y},t_{z}$ of the pose; (**d**) angle error θ; (**e**) gap error τ; and (**f**) offset error ϵ of the optical layer.

We also evaluated the same dataset with additional noise to verify the robustness of the proposed algorithm in a noisy environment. We added Gaussian noise from signal-noise-ratio (SNR) 0–10 to all the generated datasets. Given the 2200 images, we also evaluated the errors of the parameters. The result of parameter estimation errors is described in Figure 9. Note, error magnitudes of the parameters related to the optical element are quite low even with a significant amount of noise. On the other hand, the translation error of the pose tends to be affected by noise levels up to values greater than 5 dB of the SNR.

### 4.2. Real System

**Figure 10:**
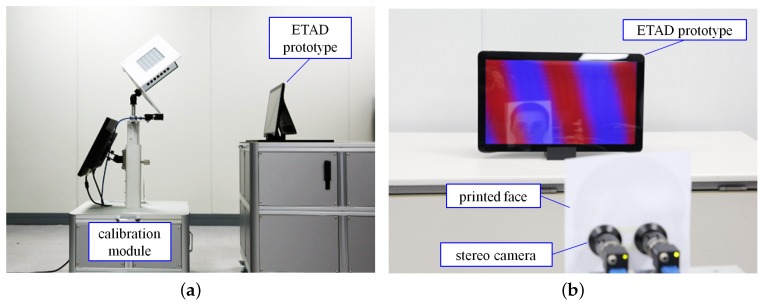
Calibration process: (**a**) calibration process using the calibration module; and (**b**) verification process using a stereo camera with a fake facial image.

We also applied the proposed method to a real-life ETAD. The proposed method was applied to a prototype of the ETAD system that included an 18.5-inch LCD panel with a resolution of 1920 × 1080. We inserted an additional parallax barrier in front of the display panel. The parameters concerned with the calibration module, i.e., external camera parameters and the pose $T_{x}^{p}$ were obtained before beginning the calibration process. Because our calibration process is fully automated, all we had to do was place the ETAD in front of the calibration module (see Figure 10a). The ETAD was connected to the calibration module through Wi-Fi. Then, the images were simultaneously captured from the onboard camera and the external camera. The image of the onboard camera was transmitted to the workstation in the calibration module. Using the PC in the module, all parameters were estimated and the results were finally transmitted to the prototype. The overall processing time from connection to writing parameters onto the tablet was less than 1.46 s. The reason behind the overall processing time being longer than the simulation processing time is that it takes into consideration the waiting time after command and image transmission.

After completing the calibration, we performed the visual quality evaluation using the calibrated parameters. To measure the view of the user, we set up stereo cameras, approximately 65 mm apart, and attach a printed image of a human face onto the lens shown in Figure 10b. The onboard camera continuously detected the eyes from the face-mimicking image, and a multiplex image was generated from a set of left and right images. We set the stereo camera system at different positions with z-axis values ranging 300–600 mm and performed the above-mentioned process. For every captured image, we adjusted the color to compensate for the difference of RGB spectra between the display and the camera.

For the measurement of the crosstalk, we encoded the views based on color, assigning red to the left, and blue to the right. Ideally, only red pixels are visible on the left image and blue pixels are visible on the right images. However, if errors of parameters are large, the views are not sufficiently separated, and a mix of both images is displayed. This effect is referred to as extrinsic crosstalk. We measured the extrinsic crosstalk according to the scheme described in [31]:

(24) $$\text{Extrinsic crosstalk}\left(\%\right)=\frac{\text{Incorrect view luminance}}{\text{Correct view luminance}}\times 100.$$

**Figure 11:**
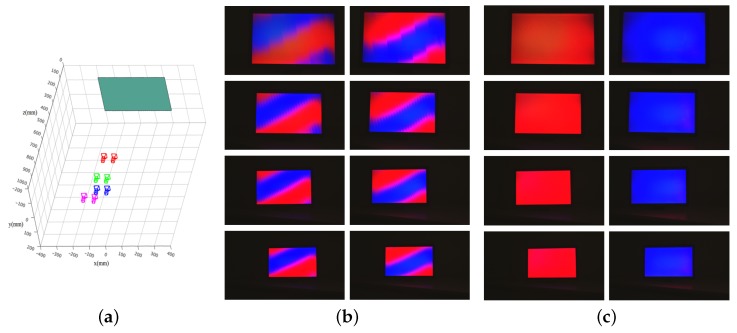
Examples of the two-view image captured at different distances: (**a**) captured positions of the stereo camera with a fake facial image; (**b**) captured images of no-calibration; and (**c**) captured images using the proposed method. The images in Rows 1–4 were captured in the positions at the test positions #1–#4, which are colored in red, green, blue, and magenta in (**a**), respectively.

We set four positions of different distances between 300 mm and 600 mm. Then, we captured 100 images at each position and analyzed the crosstalk. Figure 11 shows the left and right captured images at different camera positions. In the case of no calibration, the left view (red) and the right view (blue) appear mixed. On the other hand, the left and right views are sufficiently separated when using the parameters from the proposed method. The results are summarized in Table 3. The average external crosstalk value was 8.32%, which is within an acceptable range for a two-view autostereoscopic display [32]. Note, the crosstalks of the no-calibration case are so large that it may be difficult for users to perceive scenes in 3D.

We also evaluated the visual quality in terms of PSNR and SSIM index. In this case, the two views of red–blue are no longer suitable because both metrics need a reference image, which has various colors and structure. We employed de facto standard datasets to evaluate stereo and multi-view image quality such as Middlebury stereo dataset [33,34], The Stanford Light Field Archive [35], MIT Synthetic Light Field Archive [36], and Disney Light Field Archive [37]. From the datasets, we selected eight images, i.e., Lego Knights, Fish, Flower, Aloe, Motorcycle, Baby3, Couch, and Bicycle1. To obtain the reference images of the selected ones, we followed the same method proposed in [21]: We first identified the view corresponding to the camera position. We then displayed the viewpoint image, which was one of the left or the right image, on the panel. This means that a 2D image was displayed on the ETAD. Then, we captured the image, which was free from artifacts caused by the other view image. The evaluation was performed in the same manner, except that we used a multiplexed image rather than a 2D image.

**Figure 12:**
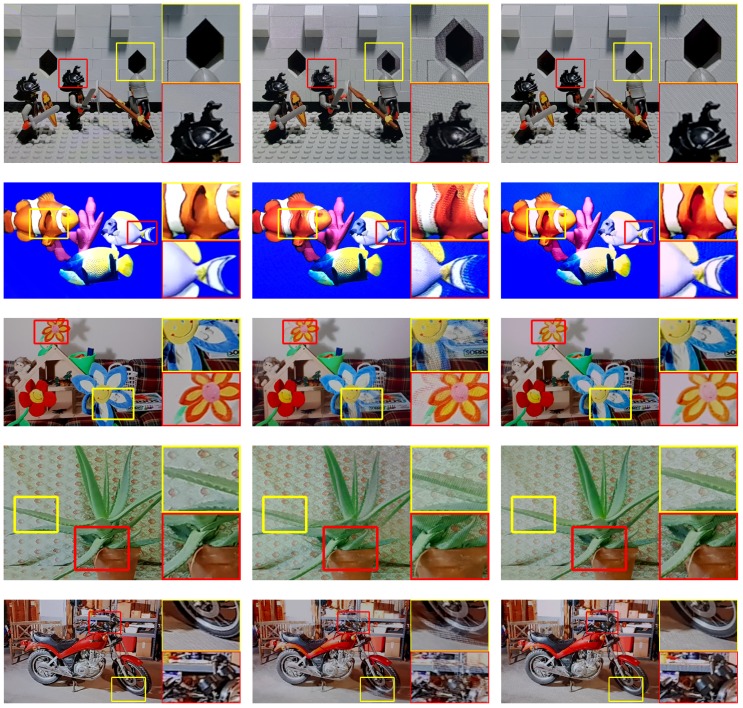

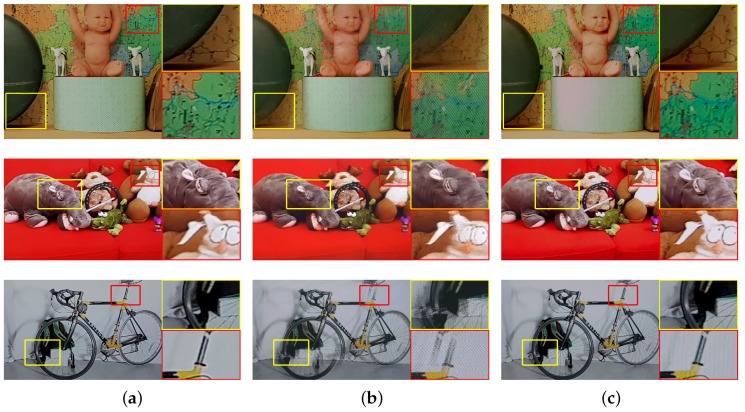
Example images observed on a real-life ETAD display. The images Lego Knights, Fish, Flower, Aloe, Motorcycle, Baby3, Couch, and Bicycle1 (from top to bottom) were rendered using the parameters from our designed method and the calibration results: (**a**) reference; (**b**) no calibration; and (**c**) calibration using the proposed method.

The results of the visual quality assessment are listed in Table 4. The gains of using the proposed method over no calibration are large (5.62 dB in PSNR, 0.2 in SSIM index on average). Figure 12 also shows more details of the results. Similar to the crosstalk evaluation results, two views are mixed in a view when rendering without calibration. In the images of the no-calibration cases, the edges in the wall bricks, the fish’s tail, the flower’s petal, the leaf of aloe, the front wheel of the motorcycle, the edge of ball, the hippo’s eye, and the front wheel of the bicycle are exposed in duplicate. However, we can verify that those artifacts almost disappear when the proposed method is used.

## 5. Conclusions

Despite the ability of the ETAD to fix issues faced by the autostereoscopic displays, the parameters of the ETAD become more complex and need higher levels of accuracy. In terms of mass production, the calibration process is essential for devices whose parameters deviate from designed ones. In this paper, we propose an efficient calibration method to estimate all variable parameters of the ETAD.

First, we determined the required parameters of the ETAD system and classified the parameters based on their ability to be misaligned during the assembly process. To fix the parameter errors, we proposed a simultaneous estimation algorithm using two images. For the automated process, we designed a calibration module comprising a pattern and an external camera. By using the module, the entire calibration process can be carried out in fixed environments, thus avoiding the need of any external manipulation. Experimental results obtained by evaluating the proposed method using a synthesis dataset show that the proposed method minimized the estimated parameter errors to within 1 s. Using a real ETAD prototype, rendering with the calibrated parameters limited the crosstalk to under 8.5%. The visual quality was also improved to 30.44% in PSNR and 40.14% in SSIM index by using the proposed method.

## Figures and Tables

**Table 1:** Device parameters for the ETAD rendering process.

Device	Parameters	Symbols	Category
onboard camera	focal length	$f_{x}$,$f_{y}$	variable
	principal point	$c_{x}$,$c_{y}$	variable
	distortion coefficient	$k_{1}$,$k_{2}$,$k_{3}$,$k_{4}$	variable
display panel	pixel width	$\mu_{w}$	constant
	pixel height	$\mu_{h}$	constant
optical element	pitch	*p*	constant
	slanted angle	θ	variable
	gap (thickness)	τ	variable
	offset	ϵ	variable
onboard camera pose	rotation	$r_{x},r_{y},r_{z}$	variable
	translation	$t_{x},t_{y},t_{z}$	variable

**Table 2:** Designed parameters and experimental results in the simulation environment.

Device	Parameter (Unit)	Designed		Estimation Errors	
		Designed Value	Variation	Mean of Absolute Error	Standard Deviation
onboard camera	$f_{x}$ (pixel/mm)	1506.9	±40.0	0.9167	0.7122
	$f_{y}$ (pixel/mm)	1506.9	±40.0	0.6045	0.4178
	$c_{x}$ (pixel)	960	-	-	-
	$c_{y}$ (pixel)	480	-	-	-
optical layer	*p* (mm)	0.01237	-	-	-
	θ (°)	12.5288	±1.0	0.0041	0.0031
	τ (mm)	0.5	±0.1	0.0252	0.0175
	ϵ (mm)	0.2	±0.1	0.0135	0.0087
onboard camera pose	$r_{x}$ (°)	0	±1.0	0.9362	0.5986
	$r_{y}$ (°)	0	±1.0	0.8596	0.6038
	$r_{z}$ (°)	0	±1.0	0.0160	0.0124
	$t_{x}$ (mm)	204.48	±5.0	0.4171	0.3226
	$t_{y}$ (mm)	−15.0	±5.0	0.1804	0.1364
	$t_{z}$ (mm)	2.0	±5.0	0.6030	0.3814

**Table 3:** Crosstalk evaluation of the ETAD using red-blue images.

Distance	No Calibration	Proposed Method
test position #1	77.5889	7.9029
test position #2	85.7855	8.0671
test position #3	91.0957	8.5187
test position #4	88.1954	8.7885
mean	85.6664	8.3193

**Table 4:** Visual quality assessment. The shown numbers are the mean and maximum deviations (in parenthesis) of the PSNR and the SSIM index over four display instances.

Name	PSNR (Standard Deviation)		SSIM (Standard Deviation)	
	No Calibration	Proposed	No Calibration	Proposed
Lego Knights	18.51 (2.46)	26.25 (0.46)	0.44 (0.02)	0.65 (0.03)
Fish	17.83 (0.64)	22.75 (0.10)	0.56 (0.02)	0.80 (0.03)
Flower	23.62 (0.36)	30.18 (0.30)	0.68 (0.01)	0.92 (0.04)
Aloe	20.55 (1.05)	29.39 (0.59)	0.48 (0.04)	0.80 (0.03)
Motorcycle	19.67 (0.19)	26.05 (0.25)	0.72 (0.01)	0.82 (0.06)
Baby3	24.06 (0.08)	27.61 (0.49)	0.47 (0.03)	0.73 (0.12)
Couch	23.34 (0.15)	29.45 (1.02)	0.67 (0.03)	0.91 (0.17)
Bicycle1	20.07 (0.25)	28.76 (0.89)	0.34 (0.12)	0.88 (0.10)

## Appendix A.

### Appendix A.1. Eye Position Estimation

Given the unit vectors $v_{l}={\left[x_{l}^{\prime },y_{l}^{\prime },1\right]}^{T}$ and $v_{r}={\left[x_{r}^{\prime },y_{r}^{\prime },1\right]}^{T}$, we can define the 3D positions of the left and right eye $e_{l}^{c},e_{r}^{c}$ as

(A1) $$e_{l}^{c}=\kappa_{l}v_{l},$$

(A2) $$e_{r}^{c}=\kappa_{r}v_{r},$$

where κ is distance to each eye. The distance of two eyes is equal to IPD ψ and defined as

(A3) $$\psi =∥\kappa_{l}v_{l}-\kappa_{r}v_{r}∥.$$

The vector passing through the two eyes is orthogonal to face normal vector ϕ and is represented as

(A4) $$(\kappa_{l}v_{l}-\kappa_{r}v_{r})\cdot \phi =0,$$

where · represents the inner product. We assumed that the viewer is watching at the center of the camera, therefore, the face normal vector has the inverse direction of the $v_{l},v_{r}$ as

(A5) $$\phi =\frac{v_{l}+v_{r}}{∥v_{l}+v_{r}∥}.$$

From Equation (A6), we obtain

(A6) $$\kappa_{l}=\frac{v_{r}\Phi }{v_{l}\Phi }\kappa_{r}.$$

By replacing with α, which is defined in Equation (6), Equation (A6) can be rewritten as

(A7) $$\kappa_{l}=\alpha \kappa_{r},$$

(A8) $$\kappa_{r}=\kappa_{l}/\alpha .$$

We insert this into Equation (A3) to obtain the distance parameter as

(A9) $$\kappa_{l}=\frac{\Psi }{∥v_{l}-v_{r}/\alpha ∥},$$

(A10) $$\kappa_{r}=\frac{\Psi }{∥\alpha v_{l}-v_{r}∥}.$$

finally, we can rewrite the eye positions (A1) and (A3) as follows,

(A11) $$e_{l}^{c}=\frac{\Psi }{∥v_{l}-v_{r}/\alpha ∥}v_{l},$$

(A12) $$e_{r}^{c}=\frac{\Psi }{∥\alpha v_{l}-v_{r}∥}v_{r}.$$
